# Polarization-Independent Optoelectronic Modulator Based on Graphene Ridge Structure

**DOI:** 10.3390/nano11102559

**Published:** 2021-09-29

**Authors:** Shiliang Guo, Xin Li, Zechen Guo, Xingtao Zhao, Shuhan Meng, Zhiquan Li

**Affiliations:** 1 School of Electrical Engineering, Yanshan University, Qinhuangdao 066004, China; guozechen148@163.com (Z.G.); zxt-81@163.com (X.Z.); 17865311232@163.com (S.M.); Lzq54@ysu.edu.cn (Z.L.); 2 School of Mathematics & Information Technology, Hebei Normal University of Science & Technology, Qinhuangdao 066004, China; lixinapple100@163.com

**Keywords:** ridge structure, polarization-independent, graphene, optoelectronic modulator

## Abstract

In this paper, we propose a polarization-independent optoelectronic modulator based on the electrical absorption effect of graphene. Firstly, we use the simulation software COMSOL Multiphysics to design the structure, and find via changing the applied voltage on both ends of the graphene that the equivalent refractive index of graphene can be changed, thus changing the light absorption capacity of the modulator. The waveguides in the transverse magnetic (TM) and transverse electric (TE) modes have almost the same extinction coefficient by making a double-layer graphene ridge structure in the center of the silicon-based waveguide, which can achieve approaching modulation depth in the TM and TE modes. At 1550 nm wavelength, the two-dimensional cross-section of the structure is analyzed by the FEM method using COMSOL Multiphysics to obtain the effective refractive index of the structure. The simulation results show that when the distance between the double-layer graphene isolation layer is *d* = 20 nm, the TE and TM modes can achieve extinction ratios up to 110 dB over the wide communication band by selecting appropriate “ON” and “OFF” switching points. The bandwidth is 173.78 GHz and the insertion loss is only 0.0338 dB.

## 1. Introduction

The modulator made of traditional photoelectric materials has been widely used in the field of optical communication [1,2,3,4]. In recent years, due to its slow modulation rate, it has been unable to meet the demand of high speed optical communication. The emergence and development of graphene technology makes it possible to solve this problem. The carrier mobility of graphene can reach 20,000 cm^2^/(V·s), and its speed is more than 100 times that of silicon [5,6,7]. Graphene also has the advantages of electrical tunability, low loss and stable chemical properties [8], so it has become a popular material for the preparation of the new generation of optical modulators. In past years, there have been many studies on graphene optoelectronic modulators. In 2004, Novoselov and Geim’s group in the UK successfully isolated monolayer graphene at room temperature for the first time by using the mechanical stripping method [9], which started the research boom into two-dimensional graphene materials [10,11,12]. In 2010, L. Ming et al. [13] covered a single layer of graphene on the upper surface of the strip-shaped silicon waveguide, and regulated the voltage through gold electrodes at both ends of graphene. The modulation depth of this device reaches 0.1 dB/μm, and the 3 dB bandwidth is 1 GHz. Furthermore, the distance between the gold electrode and the main waveguide is set to 500 nm to avoid the current affecting the optical field distribution in the main waveguide. By applying a voltage to both ends of graphene, the effective refractive index of graphene can be changed to achieve the modulation of incident light. However, due to the low light absorption rate of monolayer graphene, its performance is poor. In 2012, the team proposed an optoelectronic modulator based on double-layer graphene [14], and the atomic layer deposition (ALD) technique was employed to conformally coat a 5 nm thick Al_2_O_3_ isolation layer to prevent potential carrier injections from the bottom graphene layer into the silicon. Two layers of graphene are attached to each gold electrode, which increases the graphene’s interaction with light and prevents free electrons from infiltrating the silicon layer. The modulation depth of the modulator reaches 0.16 dB/μm, but the 3 dB bandwidth does not increase, mainly because the RC time constant of the modulator remains unchanged. The modulator structure of this study provides the basis for our design. Kim et al. placed graphene in the area of the waveguide with the strongest light intensity and used an hBN spacer with a thickness of 7 nm to separate graphene layers and between graphene and silicon waveguide, enhancing graphene-light interaction and modulation depth [15]. Our group also fabricated a micro-ring optoelectronic modulator using double-layer graphene, with an oxide layer thickness of 10 nm between double-layer graphene [16,17].

At present, most of the current graphene-based optoelectronic modulators are polarization-dependent [18,19,20], which can only regulate a specific transverse magnetic (TM) or transverse electric (TE) mode. However, in the actual optical communication system, the polarization state of the optical signal changes randomly, in this case, the polarization-dependent optoelectronic modulators will inevitably cause polarization-sensitive loss [21,22,23]. Therefore, the research on the polarization-insensitive optoelectronic modulator is of practical significance. In 2017, M.K. Shah et al. [24] transferred two graphene sheets with 90-degree bend to the inside of the waveguide core layer by using the principle of taking both the horizontal and vertical directions of the graphene layer into consideration, thereby making a graphene-based polarization-independent light modulator. Their research results show that, the extinction ratio (ER) of the device is 18.87 dB, the insertion loss is 2.32 dB, the quality factor is 8.14, and the polarization independence of the device is guaranteed at the same time. In the same year, Shengwei Ye et al. [25] deposited a sloped trapezoidal double-layer graphene layer in the middle of the silicon waveguide, so that the graphene can interact with the polarized light field in both the horizontal and vertical directions, and also achieved polarization-independent modulation. When the working wavelength of the modulator is 1550 nm, the extinction ratio is up to about 45 dB, and the 3 dB bandwidth can reach 69.8 GHz. Compared with this structure, the modulator proposed in this paper has a higher extinction ratio and lower insertion loss. In addition, the structure is simpler and the manufacturing difficulty of the device is reduced.

This paper presents a polarization-independent optoelectronic modulator based on the graphene absorption effect and simulated by COMSOL Multiphysics software. By transferring the graphene to the upper surface of the ridge waveguide and depositing the silicon material of a certain thickness, the double-layer graphene is positioned at the center of the silicon waveguide, and the interaction between the graphene and the light field is enhanced. Because graphene can fully interact with the light field in both the horizontal and vertical directions, the TM and TE modes have the same extinction coefficient, achieving the purpose of polarization independence. The designed modulator in this paper provides a theoretical foundation for the further development of optical communication

## 2. Structure Design and Theory Analysis

### 2.1. The Structure Design

**Figure 1:**
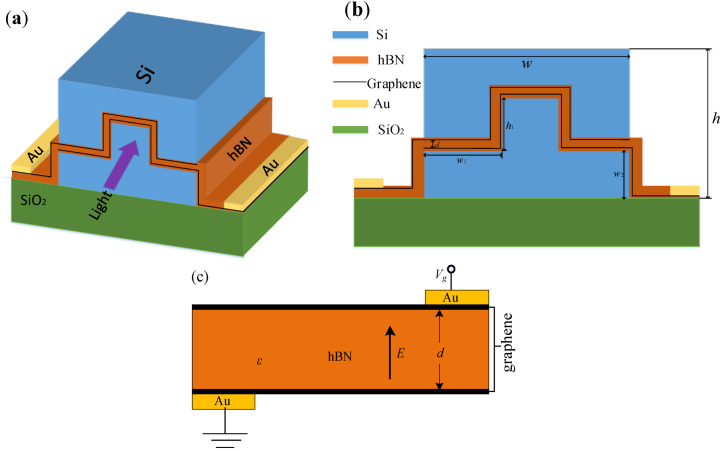
The structure of the polarization-independent graphene optoelectronic modulator (**a**) three-dimensional structure diagram (**b**) schematic diagram of waveguide cross-section structure (**c**) the diagram of applied voltage modulation.

The structure of the polarization-independent optoelectronic modulator based on the graphene ridge structure proposed in this paper is shown in Figure 1. First, the silicon waveguide with the width of 660 nm and the thickness of 340 nm is epitaxially grown on the silica-on-silicon substrate. Using the Si etching technique, the Si waveguide is etched into $w_{1}$ = 220 nm and $h_{1}$ = 170 nm. Two CVD-grown graphene flakes are transferred, which are separated by a 40 nm thick hBN isolation layer. Then another silicon waveguide was epitaxially grown on the surface, and a rectangular silicon graphene waveguide (SGW) with the height of 510 nm and the width of 660 nm was formed by electron beam lithography. The 5 nm-thick hBN isolation layer separates the upper and lower graphene sheets from the silicon to prevent carriers from being injected into the silicon. The epitaxial upper graphene sheet is connected to the Au metal electrode on the left side of the waveguide, and the lower graphene sheet is connected to the Au metal electrode on the right end of the silicon waveguide to form a double-layer graphene modulation region, the diagram of applied voltage modulation is shown in Figure 1c.

Double-layer graphene uses the hBN with stable chemical properties and high refractive index as the material of the isolation layer. By selecting the appropriate thickness of the isolation layer, both the high extinction ratio and the wide modulation bandwidth can be achieved. When the graphene is placed horizontally in the center of the silicon waveguide, the graphene mainly interacts with the TE mode light in the waveguide; when the graphene is placed vertically in the center of the silicon waveguide, the graphene mainly interacts with the TM mode light. Therefore, when the graphene has both horizontal and vertical coverage, the TM and TE mode light can be modulated at the same time. By choosing appropriate values for the length of the graphene in the horizontal and vertical directions, it is possible to achieve polarization-independent optoelectronic modulation.

### 2.2. The Optoelectronic Properties of Graphene

The graphene conductivity is the concentrated expression of its optoelectronic properties, which is defined by the Kubo equation [26].

(1) $$\sigma (\omega )=\sigma^{\mathrm{intra}}+\sigma^{\mathrm{inter}}$$

(2) $$\sigma^{\mathrm{intra}}=\frac{2ie^{2}k_{B}T}{\pi \hbar^{2}\left(\omega +i\tau^{-1}\right)}\ln\left[2\cosh\left(\frac{\mu_{c}}{2k_{B}T}\right)\right]$$

(3) $$\sigma^{\mathrm{inter}}=\frac{e^{2}i\left(\omega +i\sigma^{-1}\right)}{4\pi k_{B}T}\int_{0}^{\infty }\frac{G\left(\xi \right)}{\hbar^{2}{\left(\omega +i\sigma^{-1}\right)}^{2}/{\left(2k_{B}T\right)}^{2}-\xi^{2}}d\xi$$

where σ(ω) is the total conductivity of graphene, $\sigma^{\mathrm{intra}}$ is the in-band conductivity of graphene, $\sigma^{\mathrm{inter}}$ is the out-band conductivity of graphene, ω is the angular frequency of the incident light, *e* is the elementary charge; ℏ is the Planck constant of the reduction; τ is the relaxation time, which is closely related to the carrier mobility *μ* and the chemical potential $\mu_{c}$.

**Figure 2:**
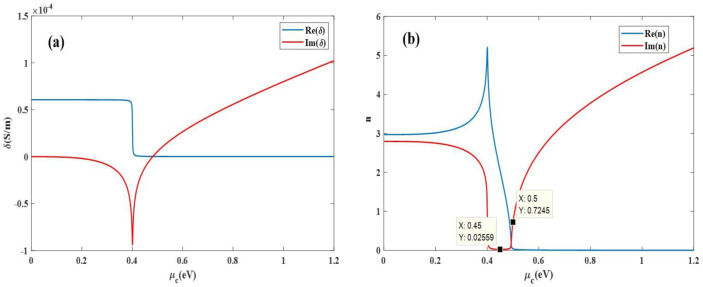
The graph of the electrical conductivity and equivalent refractive index with the graphene chemical potential. (**a**) Conductivity. (**b**) Equivalent refractive index.

Figure 2a shows the graph of the graphene’s electrical conductivity as the function of chemical potential. The graph shows that the real value of graphene’s electrical conductivity decreases from 0.6 to 0 when the chemical potential is 0.40 eV. The imaginary value decreases from 0 to −0.9 when the chemical potential is around 0.40 eV. According to scientific literature [27,28,29], the equivalent permittivity of graphene can be obtained from the conductivity, as shown in Equation (4):

(4) $$\varepsilon \left(\omega \right)=1+\frac{i\delta \left(\omega \right)}{\omega \varepsilon_{0}d_{1}}$$

where δ(ω) is the total conductivity of graphene, ω is the angular frequency of the incident light, $\varepsilon_{0}$ is the absolute dielectric constant of the vacuum, $d_{1}=$ 0.7 nm is the thickness of the single-layer grapheme [30]. Figure 2b shows the relationship between the equivalent refractive index of graphene and the chemical potential. When the chemical potential is less than 0.40 eV, the real part of the equivalent refractive index of graphene increases as the chemical potential increases. It decreases rapidly after reaching the maximum when the chemical potential is 0.40 eV, and gradually stabilizes after $\mu_{c}=$ 0.50 eV. With the increase of the graphene chemical potential, the imaginary part of the equivalent refractive index gradually decreases and reaches the minimum value at $\mu_{c}=$ 0.45 eV, and then gradually increases after the period of stabilization. The real part N of the equivalent refractive index of the graphene depends on the propagation speed of the light wave in the waveguide; the imaginary part α depends on the attenuation of the light wave as it propagates through the waveguide, which is called the extinction coefficient.

Graphene is a two-dimensional material with zero band gap, which can respond in the wavelength range of visible to near-infrared light, thus escaping the “long wave limit” of traditional detectors. When the incident light energy is weak, the light absorption properties of graphene are mainly determined by the carrier concentration of graphene. That is because the graphene’s ability to absorb light depends on the Fermi level of the graphene. When the bias voltage is applied across the graphene, it causes the change in the carrier concentration and the Fermi level [31]. This results in changes in the in-band and inter-band absorbance of graphene, which affects the light absorption of graphene and achieves optoelectronic modulation. The relationship between the driving voltage and the chemical potential of graphene is [32]:

(5) $$\mu_{c}=\hbar \nu_{F}\sqrt{\pi \frac{\varepsilon_{0}\varepsilon_{r}V_{g}}{de}}$$

where ℏ is the reduced Planck constant, $v_{F}\approx 10^{6}\;{\mathrm{ms}}^{-1}$ is the Fermi velocity, $\varepsilon_{0}=8.85\times 10^{-12}\;F/m$ is the absolute dielectric constant in vacuum, $\varepsilon_{r}$ is the dielectric constant of the isolation medium between the two layers of graphene, d is the distance between two layers of graphene, $V_{g}$ is the applied driving voltage. When the incident light intensity exceeds a certain critical value, the absorption of light by graphene does not increase with the increase of light intensity, but remains at a stable light absorption level. Therefore, the influence of optical power on optical modulation should be considered during operation.

## 3. Simulation Results and Analysis

Based on the finite element method, the mode analysis module of COMSOL software is used to simulate the two-dimensional cross-section of the modulator in Figure 1b, and the effective mode index of the structure is obtained. As shown in Figure 1b, the total width of the silicon waveguide of the modulator is w and the total height is h; the length of the three sections of graphene placed horizontally in the middle of the silicon waveguide is $w_{1}$ and the height of the two sections of graphene placed vertically is $h_{1}$; the width of the isolation layer between the two layers of graphene is *d*; the distance between horizontal graphene and the bottom surface of the silicon waveguide is $w_{2}$. The refractive index of the material used in this device is as follows: $n_{s_{i}o_{2}}=1.44$, $n_{s_{i}}=3.47$, $n_{hBN}=1.98$. The equivalent refractive index of graphene is calculated from the previous chapter. The specific values of other parameters are as follows: w= 660 nm, h= 510 nm, d= 20 nm, $w_{1}=$ 220 nm, $w_{2}=$ 170 nm, $h_{1}=$ 170 nm.

**Figure 3:**
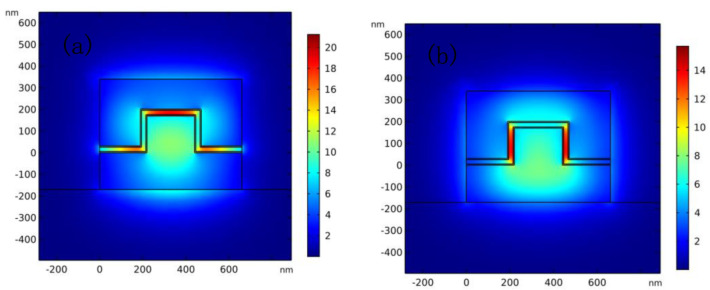
The mode field distribution in the waveguide. (**a**) The TM mode field distribution (**b**) The TE mode field distribution.

Figure 3a,b are respectively the electric field diagrams of the TM and TE modes. It can be seen from Figure 3a that the light field in the TM mode mainly interacts with horizontally placed graphene, and most of the light is confined between the two layers of graphene. The light field in the TE mode mainly is confined in vertically placed graphene as shown in Figure 3b. Polarization-independent optoelectronic modulation can be achieved through the two parts of graphene. Placing the graphene ridge structure in the center of the waveguide enhances the interaction between the light and graphene. Under the premise of ensuring low polarization tolerance, the ER of the modulators in TM and TE modes are improved. Although a small amount of light leakage occurs in the vertical and horizontal light fields, resulting in diffraction effect, the effect on the overall polarization characteristics of the light modulator is negligible.

**Figure 4:**
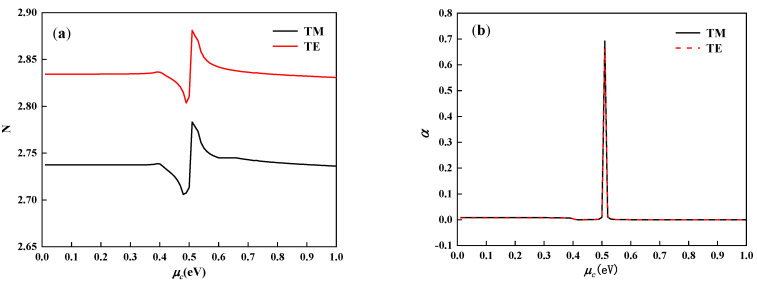
The real and imaginary parts of the effective refractive index changes in transverse magnetic (TM) and transverse electric (TE) modes at different graphene chemical potentials. (**a**) The effective refractive index real part. (**b**) The imaginary part of effective refractive index.

At 1550 nm light wavelength, the real and imaginary parts of the effective refractive index with chemical potential can be obtained by using the COMSOL software. As shown in Figure 4a, as the chemical potential increases, the real part N of the effective refractive index of the structures in the TM and TE modes has a nearly uniform trend of change. As shown in Figure 4b, when the chemical potential changes, the imaginary part *α* of the effective refractive index in the TM and TE modes is almost equal. When $\mu_{c}=0.51\;\mathrm{eV}$, the $\alpha_{TM}$ in TM mode is 0.692, and the $\alpha_{TE}$ in TE mode is 0.665. At this time, the light absorption in the two modes is the strongest, which can be used as the “OFF” point of the modulator. Meanwhile, the maximum difference between the $\alpha_{TM}$ and $\alpha_{TE}$ is 0.027, which is the small value relative to the peak. It shows that the device has almost the same modulation ability for the TM and TE modes. When $\mu_{c}=$ 0.61 eV, $\alpha_{TM}$ and $\alpha_{TE}$ are equal to $1.1789\times 10^{-4}$ and $8.3459\times 10^{-5}$, respectively. At this time, most of the light field can pass through the modulator, and this point is used as the “ON” point of the device.

**Figure 5:**
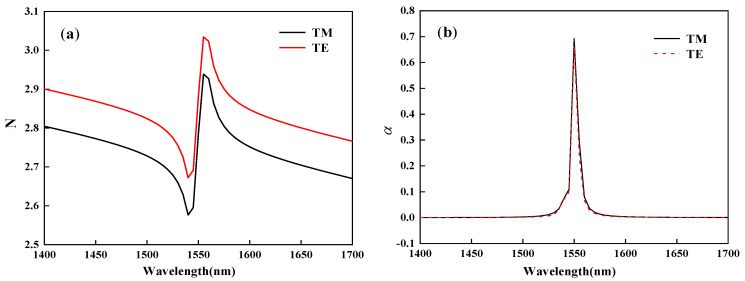
The real and imaginary parts of the effective refractive index changes in TE and TM modes at different wavelength (**a**) The real part of effective refractive index (**b**) The imaginary part of effective refractive index.

When the chemical potential is equal to 0.51 eV and the wavelength of the incident light is changed from 1400 nm to 1700 nm, the variation of the real part N and imaginary part *α* of the effective refractive index with wavelength is shown in Figure 5. As shown in Figure 5a, $N_{TM}$ and $N_{TE}$ have almost the same change trend. As shown in Figure 5b, when the wavelength changes, the difference between the value of $\alpha_{TM}$ and $\alpha_{TE}$ is extremely small. The maximum difference between the $\alpha_{TM}$ and $\alpha_{TE}$ is only 0.027, and both take the maximum value at the wavelengths equal to 1550 nm.

**Figure 6:**
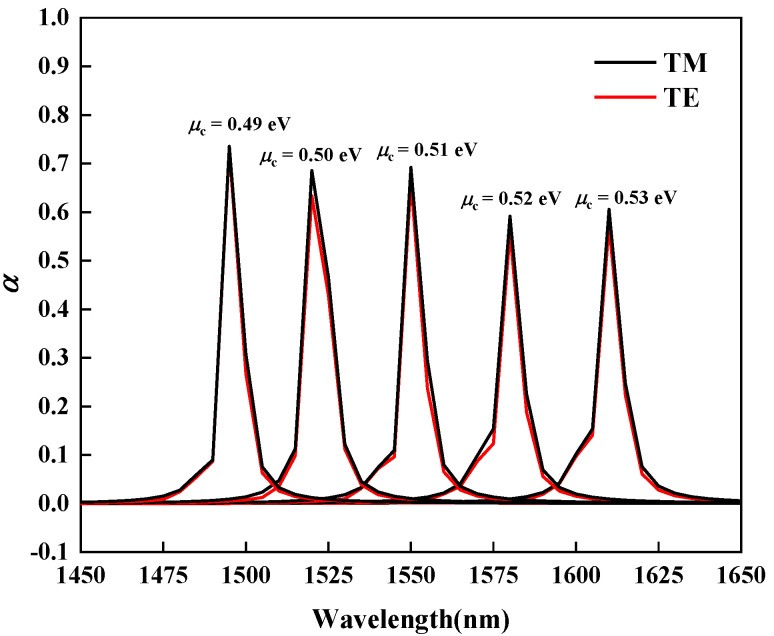
When $\mu_{c}$ is equal to 0.49 eV, 0.50 eV, 0.51 eV, 0.52 eV, 0.53 eV, the variation of the imaginary part of effective refractive index for both TE and TM modes at different wavelength.

As shown in Figure 6, when $\mu_{c}$ is equal to 0.49 eV, 0.50 eV, 0.51 eV, 0.52 eV, and 0.53 eV, the wavelength taken to the extinction coefficient $\alpha_{\max}$ also increases as the chemical potential increases. By choosing different chemical potentials, the device can maintain the high extinction coefficient over the wide range of wavelengths. At the same time, the values of $\alpha_{TM}$ and $\alpha_{TE}$ are almost equal for the same chemical potential, which again confirms the good polarization independence of the device.

## 4. The Device Performance Parameters

### 4.1. The Extinction Ratio

The extinction ratio is the important parameter of an optoelectronic modulator and the index to measure its switching performance. The expression of the ER of the modulator proposed in this paper is shown in Equation (6):

(6) $$ER=10\;\times \;\log_{10}\left(\frac{P_{ON}}{P_{OFF}}\right)=10\;\times \;\log_{10}\left(\frac{e^{-4\pi \alpha_{ON}L}}{e^{-4\pi \alpha_{OFF}L}}\right)$$

where $P_{ON}$ and $P_{OFF}$ represent the power of the modulator when it is in the “ON” and “OFF” states; *L* is the effective length of the modulator; $\alpha_{ON}$ and $\alpha_{OFF}$ are the extinction coefficient when the modulator is respectively in the “ON” and “OFF” states, which is the imaginary part of the effective refractive index of the modulator.

**Figure 7:**
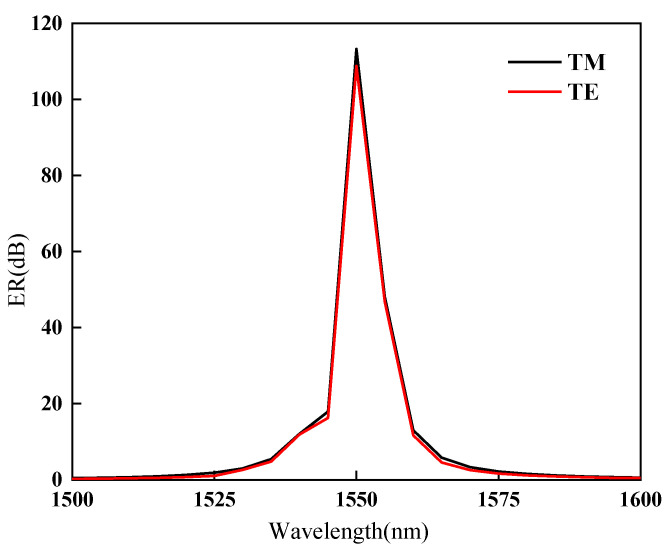
When $\mu_{c}=$ 0.61 eV is selected as “ON” state and $\mu_{c}=$ 0.51 eV is the “OFF” state, the change of the ER with wavelength for both TM and TE modes is calculated.

As shown in Figure 4b, when the chemical potential equals to 0.61 eV, the light modulator has the low extinction coefficient value, and the extinction coefficient α at this time is selected as $\alpha_{ON}$. When the chemical potential equals to 0.51 eV, the light modulator has the highest extinction coefficient value, and the extinction coefficient α is selected as $\alpha_{OFF}$. The article selects the modulation length *L* = 3 μm, changes the incident wavelength λ, and calculates the change of the ER value for both TM and TE modes with the incident light wavelength as shown in Figure 7. When the operating frequency is around 1550 nm, the modulator has the maximum ER of 110 dB, which is much larger than the previously proposed structure [24]. At the same time, almost the same ER of TM and TE mode is guaranteed. The calculation results show that the ER gap between TM and TE modes is always less than 4.46 dB, which is much smaller than the previously proposed structure. This can make the polarization tolerance of the optical modulator lower, so as to obtain a better polarization non-light modulation effect [25]. As shown in Figure 6, when $\mu_{c}$ is equal to 0.49 eV, 0.50 eV, 0.51 eV, 0.52 eV, and 0.53 eV, the high extinction coefficient $\alpha_{OFF}$ can be obtained in the wavelength range of 1450 nm to 1650 nm. The high ER is thus achieved in this wavelength range by fixing $\alpha_{ON}$ and changing different $\alpha_{OFF}$. It has also a better working bandwidth than the previously proposed modulator [25]. In summary, the optical modulator proposed in this paper can achieve good polarization-independent optoelectronic modulation in a wide range of wavelengths.

### 4.2. The Modulation Bandwidth

The important parameter for measuring the modulation rate of an optical modulator is the modulation bandwidth The factors that determine the modulation bandwidth of graphene optical modulators are mainly the equivalent capacitance and equivalent resistance of the modulator [33,34], as shown in Equation (7):

(7) $$f_{3\mathrm{dB}}=\frac{1}{2\pi RC}$$

where *R* is the equivalent resistance of the device, mainly including the resistance *Rg* of the graphene itself and the contact resistance *Rc* due to the contact between the metal electrode and graphene. Since the resistivity of the graphene is very small, the resistance *Rg* is negligible. Only the resistance *Rc* is considered in the calculation. Its value is generally $R_{c}=$ 400 Ω, and the equivalent resistance of the double-layer graphene modulator is calculated to be R= 266.67 Ω.

**Figure 8:**
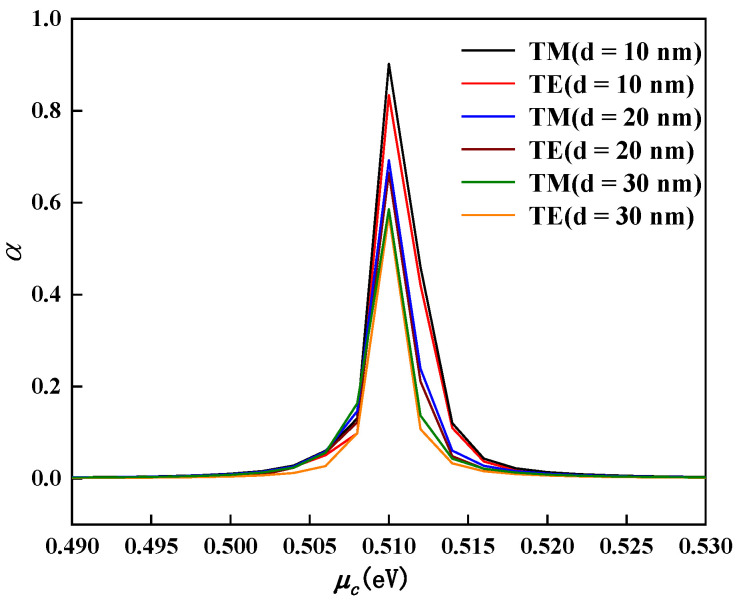
When the thickness d of the isolation layer is equal to 10 nm, 20 nm, 30 nm, the imaginary part of the effective refractive index changes with the chemical potential.

The equivalent capacitance of the device is *C*, mainly including the parallel plate capacitance *C*_P_ formed by the intermediate isolation medium, and its calculation equation is as follows:

(8) $$C_{P}=\frac{\varepsilon_{0}\varepsilon_{r}S}{d}$$

where $\varepsilon_{0}$ is the absolute dielectric constant of the vacuum, $\varepsilon_{r}$ is the relative dielectric constant of the isolation medium hBN, *S* is the area of the plate capacitor, *S = wL*, *w* = 660 nm is the width of the active region, *L* = 3μm is the length of the active region, *d* is the thickness of the double-layer graphene insulation medium. When $\varepsilon_{r}$ remains unchanged, *d* is proportional to $f_{3dB}$. However, when *d* is changed, the modulator’s extinction coefficient *α* will also change. Figure 8 shows the change of the *α* with the chemical potential in the TM and TE modes at different separation layer distances *d*. As shown in Figure 8, *d* is inversely proportional to the peak value of *α*. This also shows that the larger *d*, the smaller the ER of the modulator. Comprehensively, this paper chose *d* = 20 nm, and then obtained $f_{3dB}=$ 173.78 GHz. The bandwidth of the bias-independent modulator previously proposed is only 69.8 GHz [24].

### 4.3. The Insertion Loss

The total optical power loss caused by adding the optical modulator to the optical transmission system is called the insertion loss of the modulator. These losses include the transmission loss and the coupling loss between the light source and the waveguide. This article only considers the effect of transmission loss on the modulator insertion loss. The definition of insertion loss is as follows:

(9) $$M=10\log\left(P_{u\max}\right)=10\log\left(e^{\frac{-8\pi \alpha_{\min}L}{\lambda }}\right)$$

where $P_{u\max}$ is the maximum value of the normalized output optical power, *α*_min_ is the minimum value of the imaginary part of the effective refractive index, which is taken as $\mu_{c}=$ 0.61 eV here, *L* = 3 μm is the effective length of the modulation, *λ* is the incident wavelength. After calculation, the insertion loss of the optoelectronic modulator is 0.0338 dB.

## 5. Conclusions

The paper presents a polarization-independent optical modulator based on the double-layer graphene ridge structure. The double-layer graphene ridge structure can interact with the light field in both vertical and horizontal directions. By changing the graphene chemical potential and operating wavelength, the COMSOL Multiphysics simulation software was used to study the effective refractive index of the modulator in TM and TE modes. Due to the electric field distribution of the TM and TE modes in the designed modulator being orthogonal, polarization-independent optoelectronic modulation can be achieved through the two parts of graphene. Placing the graphene ridge structure in the center of the waveguide enhances the interaction between the light and graphene. Under the premise of ensuring low polarization tolerance, the ER of the modulators in TM and TE modes are improved. The results show that: when the graphene chemical potential changes, the imaginary parts of the equivalent refractive index in TE and TM modes have almost equal values, and both reach the maximum value when $\mu_{c}=$ 0.51 eV. At the same time, the maximum difference between $\alpha_{TM}$ and $\alpha_{TE}$ is only 0.027, which achieves the polarization-independent modulation requirements of the modulator. When the distance between the double-layer graphene isolation layer is *d* = 20 nm, the TE and TM modes can achieve extinction ratios up to 110 dB over the wide communication band by selecting appropriate “ON” and “OFF” switching points. The bandwidth is 173.78 GHz and the insertion loss is only 0.0338 dB. The proposed modulator provides the important theoretical reference for the development of large-scale integration of optoelectronic communication devices.

## References

[1] Palushani E., Galili M., Mulvad H.C., Jeppesen P. (2010). 640 Gbit/s and 1.28 Tbit/s polarisation insensitive all optical wavelength conversion. Opt. Express.

[2] Tu X., Liow T.Y., Song J., Luo X., Fang Q., Yu M., Lo G.Q. (2013). 50-Gb/s silicon optical modulator with traveling-wave electrodes. Opt. Express.

[3] Liao Y., Zhou H., Meng Z. (2009). Modulation efficiency of a LiNbO_3_ waveguide electro-optic intensity modulator operating at high microwave frequency. Opt. Lett..

[4] Tang Y., Chen H.W., Jain S., Peters J.D., Bowers J.E. (2011). 50 Gb/s hybrid silicon traveling-wave electroabsorption modulator. Opt. Express.

[5] Sun Z., Martinez A., Wang F. (2016). Optical modulators with 2D layered materials. Nat. Photonics.

[6] Cheng J., Fan F., Chang S. (2019). Recent progress on graphene-functionalized metasurfaces for tunable phase and polarization control. Nanomaterials.

[7] Zhou M., Zhou C., Luo K., Li W., Liu J., Liu Z., Wu Z. (2021). Ultrawide bandwidth and sensitive electro-optic modulator based on a graphene nanoelectromechanical system with superlubricity. Carbon.

[8] Bae S., Kim H., Lee Y., Xu X., Park J.S., Zheng Y., Balakrishnan J., Lei T., Kim H.R., Song Y.I. (2010). Roll-to-roll production of 30-inch graphene films for transparent electrodes. Nat. Nanotechol..

[9] Novoselov K.S., Geim A.K., Morozov S.V., Jiang D., Katsnelson M.I., Grigorieva I.V., Dubonos S.V., Firsov A.A. (2005). Two-dimensional gas of massless dirac fermions in graphene. Nature.

[10] Geim A.K. (2009). Graphene: Status and prospects. Science.

[11] Novoselov K.S., Geim A.K., Morozov S.V., Jiang D., Zhang Y., Dubonos S.V., Grigorieva I.V., Firsov A.A. (2004). Electric field effect in atomically thin carbon films. Science.

[12] Zhu B., Ren G., Cryan M.J., Gao Y., Lian Y., Wang J., Wan C., Jian S. (2016). Two-dimensional analogies to frequency-selective surfaces (FSS) on the graphene sheet. Plasmonics.

[13] Liu M., Yin X., Ulin-Avila E., Geng B., Zentgraf T., Ju L., Wang F., Zhang X. (2011). A graphene-based broadband optical modulator. Nature.

[14] Liu M., Yin X., Zhang X. (2012). Double-layer graphene optical modulator. Nano Lett..

[15] Kim K., Choi J.Y., Kim T., Cho S.H., Chung H.J. (2011). A role for graphene in silicon-based semiconductor devices. Nature.

[16] Li Z., Bai L., Li X., Gu E., Niu L., Zhang X. (2018). U-shaped micro-ring graphene electro-optic modulator. Opt. Commun..

[17] Li Z., Bai L., Gu E., Xie R., Li X., Li W. (2018). TBOR-shaped electro-optic modulator with dual output based on double-layer graphene. Phys. Lett. A.

[18] Ye S., Wang Z., Tang L., Zhang Y., Lu R., Liu Y. (2014). Electro-absorption optical modulator using dual-graphene-on-graphene configuration. Opt. Express.

[19] Phare C.T., Daniel Lee Y.H., Cardenas J., Lipson M. (2015). Graphene electro-optic modulator with 30GHz bandwidth. Nat. Photonics.

[20] Sun H., Zhao L., Dai J., Liang Y., Guo J., Meng H., Liu H., Dai Q., Wei Z. (2020). Broadband filter and adjustable extinction ratio modulator based on metal-graphene hybrid metamaterials. Nanomaterials.

[21] Yang Z., Lu R., Wang Y., Cai S., Zhang Y., Wang X., Liu Y. (2019). A fabrication-friendly graphene-based polarization insensitive optical modulator. Optik.

[22] Hu X., Wang J. (2018). Design of graphene-based polarization-insensitive optical modulator. Nanophotonics.

[23] Zhang S., Li Z., Xing F. (2020). Review of polarization optical devices based on graphene materials. Int. J. Mol. Sci..

[24] Shah M.K., Lu R., Peng D., Ma Y., Ye S., Zhang Y., Zhang Z., Liu Y. (2017). Graphene-assisted polarization-insensitive electro-absorption optical modulator. IEEE Trans. Nanotechnol..

[25] Ye S., Liang D., Lu R., Shah M.K., Zou X., Yuan F., Yang F., Liu Y. (2016). Polarization-independent modulator by partly tilted graphene-induced electro-absorption effect. IEEE Photonic Technol. Lett..

[26] Gosciniak J., Tan D.T. (2013). Theoretical investigation of graphene-based photonic modulators. Sci. Rep..

[27] Liu J.P., Zhai X., Wang L.L., Li H.J., Xie F., Xia S.X., Shang X., Luo X. (2016). Graphene-based long-range spp. hybrid waveguide with ultra-long propagation length in mid-infrared range. Opt. Express.

[28] Chang Z., Chiang K.S. (2016). Experimental verification of optical models of graphene with multimode slab waveguides. Opt. Lett..

[29] Hao R., Du W., Li E.P., Chen H.S. (2015). Graphene assisted TE/TM-independent polarizer based on mach–zehnder interferometer. IEEE Photonic Technol. Lett..

[30] Wang Z., Zhou M., Lin X., Liu H., Wang H., Yu F., Lin S., Li E., Chen H. (2014). A circuit method to integrate metamaterial and graphene in absorber design. Opt. Commun..

[31] Kim J., Son H., Cho D.J., Geng B., Regan W., Shi S., Kim K., Zettl A., Shen Y.R., Wang F. (2012). Electrical control of optical plasmon resonance with graphene. Nano Lett..

[32] Alexander Y.Z., Fei Y., Jason C.R., Ertugrul C. (2014). Cavity-enhanced mid-infrared absorption in perforated graphene. J. Nanophotonics.

[33] Xia F., Perebeinos V., Lin Y.M., Wu Y., Avouris P. (2011). The origins and limits of metal–graphene junction resistance. Nat. Nanotechnol..

[34] Taghi A.M., Zaharah J., Aziziah A.N., Hossein F.A., Razali I. (2010). Graphene nanoribbon conductance model in parabolic band structure. J. Nanomater..

